# Effects of Transcranial Stimulation With Direct and Alternating Current on Resting-State Functional Connectivity: An Exploratory Study Simultaneously Combining Stimulation and Multiband Functional Magnetic Resonance Imaging

**DOI:** 10.3389/fnhum.2019.00474

**Published:** 2020-02-05

**Authors:** Marine Mondino, Sukhmanjit Ghumman, Claire Gane, Emmanuelle Renauld, Kevin Whittingstall, Shirley Fecteau

**Affiliations:** ^1^Department of Psychiatry and Neurosciences, Medical School, Université Laval, CERVO Brain Research Center, Centre Intégré Universitaire en Santé et Services Sociaux de la Capitale-Nationale, Quebec City, QC, Canada; ^2^Department of Radiology, Faculty of Medicine and Health Science, University of Sherbrooke, Sherbrooke, QC, Canada

**Keywords:** tDCS–transcranial direct current stimulation, tACS (transcranial alternating current stimulation), fMRI–functional magnetic resonance imaging, functional connectivity, fronto-parietal connectivity

## Abstract

**Background**: Transcranial stimulation with direct (tDCS) and alternating current (tACS) has increasingly gained interest in various fields, from cognitive neuroscience to clinical investigations. Transcranial current stimulation used alone may modulate brain activity that consequently influences behaviors, without providing information on potentially induced brain activity changes. The combination of transcranial current stimulation and functional magnetic resonance imaging (fMRI) may help to address this. This exploratory study investigated instantaneous and subsequent effects of tDCS and tACS on resting-state functional connectivity (rsFC) in healthy adults.

**Methods**: We conducted a randomized crossover study with 15 healthy subjects receiving three stimulation conditions (tDCS, tACS, and sham) on separate days. Stimulation was applied over the left and right dorsolateral prefrontal cortex (DLPFC) for 30 min (1 mA). rsFC of the targeted prefrontal areas was assessed before, during, and after stimulation using multiband fMRI and using left and right DLPFC as seeds.

**Results**: Both tDCS and tACS increased rsFC during and after the stimulation period, as compared to sham. tDCS-induced changes were observed between the left DLPFC and bilateral parietal regions at the junction of the superior parietal and the inferior parietal lobules. tACS-induced changes were observed between the left DLPFC and the right inferior parietal lobule.

**Conclusion**: Overall, these results suggest that a single session with a low dose, 1 mA, of tDCS or tACS can cause changes in fronto-parietal connectivity that occur rapidly, that is, within the first 15 min. Although exploratory, this work contributes to the discussion of the potential of transcranial current stimulation to modulate resting-state networks and the interest of combining transcranial current stimulation with neuroimaging to identify these changes.

## Introduction

Noninvasive transcranial stimulation (tCS) techniques such as transcranial direct current stimulation (tDCS) and transcranial alternating current stimulation (tACS) are increasingly used in clinical and cognitive science. These techniques consist in the application of low-intensity constant or alternating electrical currents (usually 1 or 2 mA) through surface electrodes placed on the scalp in order to modulate brain activity. tDCS is thought to modulate neuronal activity by shifting resting membrane potential towards either depolarization or hyperpolarization (Nitsche et al., 2008) and tACS to modulate brain oscillations by entraining endogenous oscillations in a frequency-dependent way (Neuling et al., 2013).

One brain region that is often targeted with tCS in clinical and cognitive studies is the dorsolateral prefrontal cortex (DLPFC). tCS applied over the DLPFC has been shown to modulate symptoms in various psychiatric disorders such as depression (Brunoni et al., 2017; Alexander et al., 2019) and cognitive functions such as memory, attention (Dedoncker et al., 2016; Hill et al., 2016), and higher-order cognitive processes such as multitasking performances (Hsu et al., 2015, 2017, 2019). More specifically, most of these studies delivered tCS at rest with the anode and cathode electrodes on both DLPFC [e.g., over F3 and F4, respectively, according to the international 10–20 EEG system; (Nasseri et al., 2015)]. Little is however known regarding the effects of tCS montages targeting both DLPFC on brain connectivity. Indeed, most studies characterizing tCS-induced brain connectivity changes targeted the primary motor cortex or only one DLPFC (e.g., anode over F3 and cathode over the right supraorbital area; for a review, see Wörsching et al., 2016). Overall, they found that tCS can induce resting-state functional connectivity (rsFC) changes locally under the electrodes as well as distal from the electrodes. Those targeting one DLPFC reported that tDCS modulated rsFC within the fronto-parietal network (Keeser et al., 2011; Peña-Gómez et al., 2012), a resting-state network involved in cognitive engagement and attention, and mix findings in regard to the default mode network (DMN; Keeser et al., 2011; Peña-Gómez et al., 2012), which is thought to reflect an intrinsic state associated with self-related processes (Buckner et al., 2008). Further, little is known on rsFC changes during stimulation. Most studies characterized rsFC before and after tCS, and to date, only few studies investigate rsFC changes during stimulation using concurrent tCS and functional magnetic resonance imaging (fMRI; Li et al., 2019). In addition, the effects of tACS applied over the DLPFC on rsFC have yet to be investigated using fMRI.

The goal of this study was thus to characterize the effects of tCS on rsFC when targeting both DLPFC. Specifically, we investigated instantaneous and subsequent tDCS- and tACS-induced rsFC changes. The main hypotheses were that tDCS and tACS, as compared to sham tCS, would modulate rsFC between the targeted prefrontal areas within a connected widespread network involving frontal and parietal areas and that these rsFC changes would occur during stimulation and persist after the end of the stimulation period, based on previous studies (Keeser et al., 2011; Peña-Gómez et al., 2012).

## Materials and Methods

### Participants

Fifteen healthy adults participated in this study (eight women; mean age ± SD: 28.7 ± 6.2 years; range: 22–42; mean years of education: 15.9 ± 2.0 years). Thirteen participants were right-handed and two were left-handed as assessed by the Edinburgh Handedness Inventory (Oldfield, 1971). None of the participants had a history of neurological or psychiatric disorder, or had any contraindication to MRI and none reported use of psychoactive medication. The study protocol was approved by the local ethics committee. All participants gave written informed consent prior to participation.

### Experimental Design

**Figure 1:**
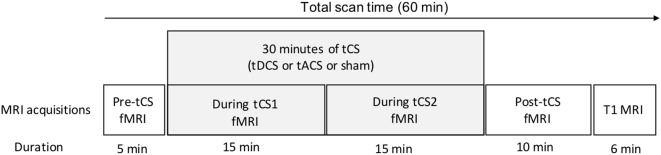
Experimental timeline of a single session. Following a 5-min pre-tCS resting-state fMRI acquisition, tCS (either active tDCS, active tACS or sham stimulation) was delivered for 30 min. During tCS, two 15-min resting-state fMRI sequences were acquired. Immediately after the end of tCS, a 10-min post-tCS resting-state fMRI sequence was acquired. Finally, an anatomical 3D T1-weighted image was acquired. MRI, magnetic resonance imaging; fMRI, functional magnetic resonance imaging; tCS, noninvasive transcranial stimulation; tDCS, transcranial direct current stimulation; tACS, transcranial alternating current stimulation.

In a sham-controlled, double-blind, crossover, within-subjects design, participants were assessed during three identical MRI sessions, separated by 1 week to minimize potential carry-over effects. During these MRI sessions, participants received active tDCS, active tACS, or sham stimulation in a counterbalanced order (six possible combinations of the stimulation order). The order of stimulation for all participants was established prior to recruitment by a researcher not involved in data collection and analyses. Please refer to Figure 1 for the experimental timeline of the MR/tCS session.

### Transcranial Direct Current and Alternating Current Stimulation (tDCS and tACS)

Stimulation was delivered by an MR-compatible battery-driven stimulator (NeuroConn GmbH, Germany) that was placed in the operator room and connected to the two 7 × 5 cm^2^ rubber electrodes through the isolated optical cable with filtering system. Electrodes were enclosed in a pair of saline-soaked sponges and fixed on the scalp with rubber bands. We used an electrode montage to target both DLPFC, that is, the anode over F3 and the cathode over F4, according to the International 10-20 EEG system. Stimulation was applied for 30 min at 1 mA intensity with ramp-up and ramp-down periods of 30 s. For tACS, an alternating current with no DC offset and with a 1-mA peak-to-peak intensity was delivered at a frequency of 6.5 Hz, corresponding to theta frequency. This frequency was chosen according to recent studies that reported cognitive effects of theta-tACS applied over the DLPFC (Sela et al., 2012; Hsu et al., 2017, 2019). Sham stimulation was performed with the same electrode montage as for the active tCS, but the current was delivered only during the first 60 s of the 30-min period. We used the “study mode” of the stimulator. Blinding was assessed in participants and tCS administrators with a visual analog scale ranging from 0 (“I think that I have received active stimulation”/(“I think that the participant received active stimulation”) to 100 (“I think that I have received sham stimulation”/“I think that the participant received sham stimulation”).

### Safety and Tolerability Assessments of tCS/Multiband fMRI

All participants completed a questionnaire before and after each stimulation session in order to assess possible stimulation-related side effects (Brunoni et al., 2011). The intensity of the following side effects was rated on a scale from 1 (none) to 4 (severe): headache, neck pain, scalp pain, cognitive changes, hearing perceptual changes, trouble concentrating, acute mood changes, and itching sensations. Participants were also asked to rate their mood on a self-reported questionnaire composed of 14 visual analog scales (VAS, rated from 0 to 100) before and after each session (i.e., calm/restless; alert/drowsy; confused/enlightened; strong/weak; satisfied/unfulfilled; worried/unconcerned; fast mind/slow mind; tense/relax; attentive/neglectful; inept/competent; happy/sad; hostile/friendly; interested/indifferent; quiet/sociable).

### MRI Acquisition Parameters

Whole-brain resting-state multi-band fMRI acquisitions were performed using a 3-T MRI scanner (Ingenia, Philips, Netherlands) and the following parameters: (T2*-sensitive echo-planar imaging, TR/TE = 900/30 ms, multi-band SENSE acceleration factor = 3, 36 axial slices, slice thickness = 3.5 mm, matrix = 64 × 64, FOV = 224 × 224 × 126 mm, voxel size = 3.5 mm^3^ isotropic). Four eyes-open fMRI sequences were acquired: one 5-min pre-stimulation sequence (330 volumes), two 15-min sequences during stimulation (during tCS1 and during tCS2, 1,000 volumes each), and one 10-min post-stimulation sequence (670 volumes). Then, an anatomical 3D T1-weighted imaging was acquired (TR/TE = 7.9/3.5 ms; FOV = 240 × 240 × 150 mm; voxel size = 1 mm^3^ isotropic).

### MRI Data Preprocessing

Anatomical data were skull-stripped and segmented into gray matter, white matter, and CSF using the automatic segmentation in FreeSurfer^1^ and then normalized to MNI using linear registration in the AFNI software (Cox, 1996; version 17.1.11). Resting-state multi-band fMRI data were preprocessed using AFNI. More specifically, the first 10 volumes of each fMRI sequence were discarded in order to attain MR stability. We then performed outlier calculation (3dToutcount), despiking (3dDespike), volume registration (3dvolreg), normalization to MNI (3dAllineate), and spatial smoothing with a 6-mm isotropic Gaussian filter (3dBlurToFWMH). Bandpass filtering in studies using relatively fast TR (as used in this study) largely suppresses the degrees of freedom in the BOLD signal and was thus not used here (please see discussions regarding bandpass filtering here: https://afni.nimh.nih.gov/afni/community/board/read.php?1,155368,155374#msg-155374). Rather, to regress out the effects of physiological noise and head motion, we used CSF/WM signals (thought to be highly contaminated by respiration), motion parameters, and their derivatives. We conducted a regression analysis using the AFNI’s 3dDeconvolve command with the following regressors: the six average motion parameters and their derivatives, white matter, and ventricle signals. Volumes with motion larger than 0.6 mm and/or in which >10% of brain voxels were determined to be outliers were removed.

### Seed-Based Functional Connectivity

Two spherical seeds of 5 mm radius were placed corresponding to the electrode placement F3 (left DLPFC; MNI coordinates *x* = −38; *y* = 25; *z* = 48) and F4 (right DLPFC; *x* = 38; *y* = 25; *z* = 48). We calculated the mean BOLD time series within each seed and then used 3dTCorr1D to generate a voxel-wise Pearson’s correlation map for each seed. We used Fisher’s *r* to *z* transformation to prepare the maps for group analysis.

### fMRI Group Analyses

Due to technical issues with the stimulation cables in the magnetic field, five participants did not receive tACS condition and two participants did not receive tDCS condition. Thus, as this work was an exploratory study investigating the effects of tDCS and tACS on rsFC and in order to avoid losing too many sensitivity to be able to detect changes, analyses were conducted separately for tDCS (*N* = 13) and tACS (*N* = 10). Two-way repeated-measures ANOVAs (AFNI program 3dANOVA3) were performed with subjects as a random effect factor and stimulation conditions (two levels: active, sham) and time points (four levels: pre-tCS, during tCS1, during tCS2, post-tCS) as fixed-effects factors. 3dClustSim (with the acf method) was used for multiple comparison correction and results were thresholded accordingly (*p* < 0.05, cluster corrected, voxel-wise *p* < 0.05, cluster size threshold of 110 voxels). To determine whether stimulation modulated rsFC, we were interested in the Stimulation × Time interaction. In case of significance, *post hoc* tests were conducted by calculating a mean Fisher’s *z* for each significant cluster (for each subject, stimulation and time condition) and then comparing the changes on rsFC from pre-tCS at each time point (during tCS1, during tCS2, post-tCS) between active and sham conditions using paired *t*-tests with SPSS-22. The anatomical label for each cluster was defined using TT_Daemon atlas implemented in AFNI.

### Blinding Efficacy and Safety Analyses

All analyses were performed using SPSS-22. Blinding ratings on VAS were attributed to two categorical responses (0–50: active; 50–100: sham) and compared between conditions using *χ*^2^ tests. Side effects were compared between stimulation conditions using paired *t*-tests. To assess potential effects on mood ratings, changes between pre- and post-stimulation assessments were compared between conditions (active, sham) using paired *t*-tests. Statistical threshold was set at *p* < 0.05.

## Results

### Safety and Tolerability of tCS/Multiband fMRI

No severe adverse effects were reported in any conditions. Between-stimulation condition comparisons revealed no difference for any side effects except for itching sensations. More precisely, participants reported more pronounced itching sensations after active tDCS (mean = 1.5; SD = 0.58) as compared to sham tCS (mean = 1.0; SD = 0.00; *T* = −3.12; *p* = 0.009). No significant differences were reported for itching sensations between active tACS and sham tCS. Regarding mood changes from pre- to post-tCS, paired *t*-tests between active and sham conditions revealed differences for the happy/sad item between tDCS (mean change = −1.2; SD = 11.2) and sham tCS (mean = 12.5; SD = 18.1; *T* = −2.21; *p* = 0.047) and between tACS (mean = −2.9; SD = 6.1) and sham tCS (mean = 12.1; SD = 20.4; *T* = −2.64; *p* = 0.027). Namely, ratings were increased towards sadness following sham tCS, whereas they remain the same after active tCS.

### Blinding Efficacy of tCS Conditions

Blinding efficacy was comparable in the three tCS conditions (tACS, tDCS, sham) for participants (*χ*^2^ = 0.24, *p* = 0.89) and tCS administrators (*χ*^2^ = 1.26, *p* = 0.53).

### Effects of tDCS on rsFC

**Figure 2:**
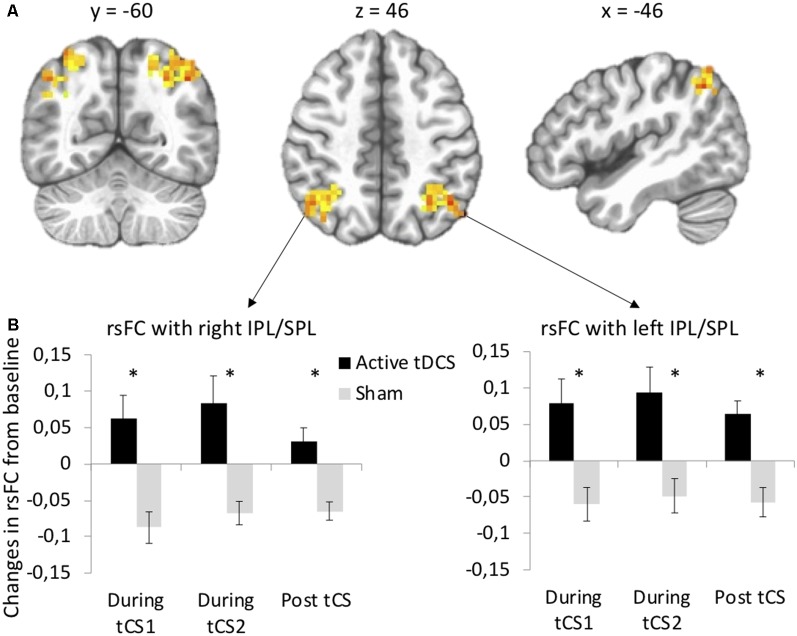
tDCS-induced changes in resting-state functional connectivity (rsFC) using the left dorsolateral prefrontal cortex (DLPFC) seed. **(A)** Clusters from the significant Stimulation (active, sham) × Time (before tDCS, during the first 15 min of tDCS, during the last 15 min of tDCS, after tDCS) interactions between the left DLPFC seed and left and right IPL/SPL. **(B)** *Post hoc* paired *t*-tests for each significant cluster between active and sham tDCS from pre-tDCS at each time point (during the first 15 min of tDCS, during the last 15 min of tDCS, after tDCS). Results are expressed as mean ± SEM. *Indicates significant differences between conditions at *p* < 0.05.

Seed-based functional connectivity analyses revealed significant Stimulation × Time interactions on rsFC between the left DLPFC seed (under the anode electrode) and two clusters: one at the junction between the left inferior (IPL, Brodmann area 40) and superior parietal lobule (SPL, Brodmann area 7) and one at the junction between the right IPL and SPL (Supplementary Table S1, Figure 2A). *Post hoc* *t*-tests indicated that active as compared to sham tDCS increased rsFC within the first 15 min of stimulation, within the last 15 min of stimulation, as well as during the 10-min period following the stimulation (Figure 2B). There was no significant interaction between Stimulation and Time on rsFC using the right DLPFC seed (under the cathode electrode).

### Effects of tACS on rsFC

**Figure 3:**
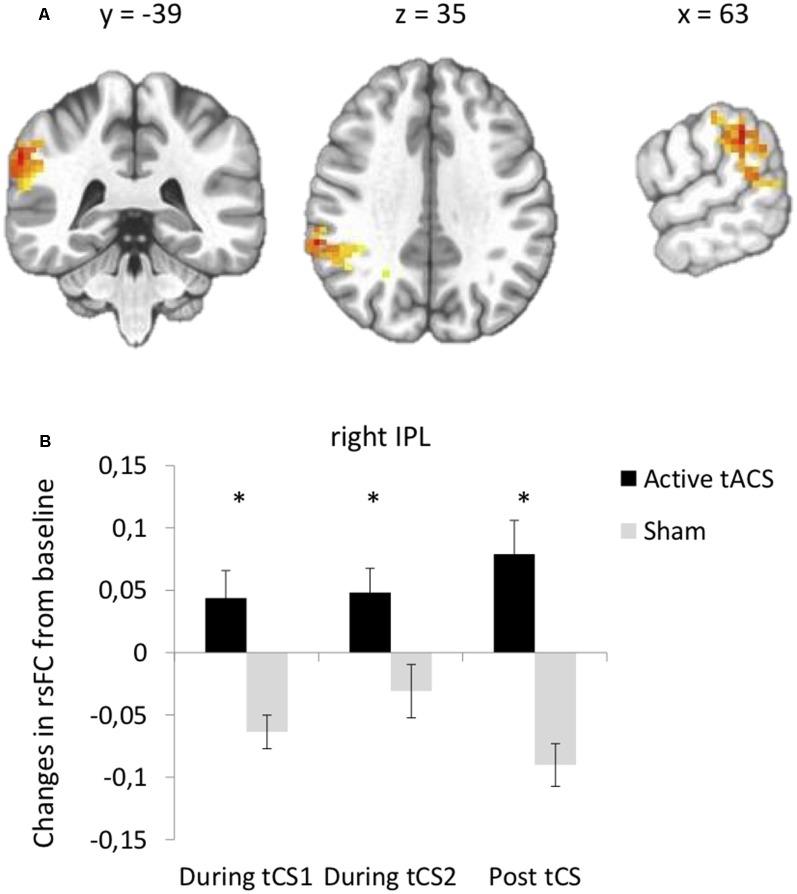
tACS-induced changes in rsFC using the left DLPFC seed. **(A)** Clusters from the significant Stimulation (active, sham) × Time (before tACS, during the first 15 min of tACS, during the last 15 min of tACS, after tACS) interactions between the left DLPFC seed and right IPL. **(B)** *Post hoc* paired *t*-tests for the significant cluster between active and sham tACS from pre-tACS at each time point (during the first 15 min of tACS, during the last 15 min of tACS, after tACS). Results are expressed as mean ± SEM. *Indicates significant differences between conditions at *p* < 0.05.

Seed-based functional connectivity analyses indicated a significant Stimulation × Time interaction on rsFC between the left DLPFC seed (under the anode electrode) and one cluster in the right IPL (Brodmann area 40, Supplementary Table S1, Figure 3A). *Post hoc* *t*-tests revealed that active as compared to sham tACS increased rsFC within the first and within the last 15 min of the stimulation period, as well as after the end of the stimulation period (Figure 3B). There was no significant interaction between Stimulation and Time on rsFC using the right DLPFC seed (under the cathode electrode).

## Discussion

In this exploratory study, we investigated whether tCS modulates rsFC, which seems to be in part driven by coherent neuronal fluctuations (Mateo et al., 2017). More specifically, we used multiband fMRI to investigate how tCS applied over both DLPFC modulates rsFC during and after the stimulation period in healthy adults. We observed that both tDCS and tACS, as compared to sham tCS, modulated rsFC, but they seem to act slightly differently.

Specifically, anodal and cathodal tDCS applied over the left and right DLPFC, respectively, as compared to sham tCS, increased rsFC between the left DLPFC seed and bilateral parietal areas at the junction of the SPL and IPL. These rsFC increases were observed during stimulation and remained significant after the stimulation period. tACS applied at 6.5 Hz over both DLPFC, as compared to sham tCS, also increased rsFC between the left DLPFC seed and the right IPL during stimulation and increased even more after the stimulation period. Overall, these results support evidence that both tDCS and tACS induced changes in fronto-parietal connectivity during and after tCS. Such rsFC modulation within the frontal and parietal regions is consistent with previous studies that investigated the effects of tDCS on rsFC (Keeser et al., 2011; Peña-Gómez et al., 2012). This rsFC modulation may be likely relevant for functions strongly associated with fronto-parietal connectivity, such as working memory and top-down control of attentional processes (Corbetta and Shulman, 2002). Another interesting finding is that there were no significant changes in rsFC when using the right DLPFC seed, that is, the area under the cathode/reference electrode of tDCS or tACS. Finally, we observed a not significant but slight decrease in rsFC for the sham tCS conditions. One could hypothesize that this decrease is linked to a time effect during resting state (for more than half an hour at rest) during which activity of the DLPFC might diminish and decorrelate from the other structures of the fronto-temporal network (here the parietal regions). It has been reported that during rest, the alpha activity increases while the BOLD signal decreases in the fronto-temporal network when simultaneously collected (Goldman et al., 2002), and the alpha activity increases while the regional cerebral blood flow decreases in the left dorsomedial PFC (Sadato et al., 1998). Here, active as compared to sham tCS might prevent this switch in rsFC of the DLPFC.

In terms of resting-state networks, tDCS and tACS modulated rsFC between regions that are known as being part of the fronto-parietal network (Vincent et al., 2008), which is also called the Central Executive Network (CEN). This network is referred to as “task-positive” because its activity increases when attention is externally oriented (Fox et al., 2005), for instance, in cognitive tasks. Of note, the strength of the fronto-parietal within-network connectivity has been shown to correlate with executive functions (Seeley et al., 2007) and IQ (Song et al., 2008; Langeslag et al., 2013). Our results of increased rsFC between fronto-parietal nodes during and after tDCS are thus consistent with the hypothesis that tCS can shift the brain resting state from an internally oriented state into a state of increased attention and readiness, which may consequently prime the brain towards an improved state to perform goal-oriented tasks (Keeser et al., 2011). This shift in brain state could be underpinned by modulation of the DMN since the two networks have been shown to share a bidirectional relation, their activity being negatively correlated in healthy controls (Greicius et al., 2003; Fox et al., 2005; Fransson, 2005) and since some evidence suggest that the fronto-parietal network may inhibit the DMN activity when noninvasive brain stimulation was delivered to one of its key nodes, the DLPFC (Chen et al., 2013). Further studies are warranted to investigate the effect of tCS on the relationship between the fronto-temporal network and the DMN. It should be noted that the functional roles of resting networks remain to be fully understood. Despite this, most agree that the level of integrity of resting networks seems to be related to some trait factors (e.g., major depressive disorders), as well as state factors (e.g., motivation), which can be clinically meaningful for some disorders (e.g., motivation to quit smoking or maintain smoking abstinence). This likely plays a role in state-dependent impact of tCS.

Investigations of the tCS effects on resting networks such as this work may provide information for future clinical studies. For instance, some recent work have identified the frontoparietal network as commonly disrupted across diagnostically distinct forms of severe affective and psychotic pathologies, namely, unipolar depression, bipolar disorder, and schizophrenia or schizoaffective disorder (Baker et al., 2014, 2019; Mulders et al., 2015). In addition, the presence of affective and psychotic illnesses has been linked to graded alterations in connectivity within this network. Here, we observed that tDCS and tACS increased rsFC within the fronto-parietal network. Both techniques might thus be potentially relevant in these conditions. However, we have to keep in mind that tCS may act differently on altered brain networks and that our findings may not necessarily be transferable to clinical populations.

Overall, tDCS and tACS induced modulation within the fronto-parietal network. Our results have however to be taken with caution due to some limitations. These findings highlight the potential of tCS to modulate brain activity when investigating rsFC of the brain areas targeted by tCS, here the left and right DLPFC. One could presume that tCS-induced rsFC changes mainly commence in areas under the electrodes but investigating changes without seeds (e.g., graph theory) would be important in future work. There might be rsFC changes due to behavioral or physiologic confounds that we cannot rule out here. Future work may consider spatial independent component or graph-based network analyses. Second, in line with this, multiband accelerated echo-planar imaging (less than 1 s), as used here, may ultimately uncover additional resting-state networks, potentially up to 10–20 networks, as it provides more precise sampling of physiologic confounds (Barkhof et al., 2014). However, of note, the multiband acquisition sequence used here disrupted tCS delivery several times, which did not occur in our previous work with concurrent fMRI/tCS. This technical issue prevented us from delivering the three stimulation conditions (tDCS, tACS, and sham) as planned and directly comparing tDCS- and tACS-induced changes in rsFC. Despite such limitation, this exploratory study demonstrated feasibility and human subject safety (but not quite tCS equipment safety!) of concomitant tCS and multiband fMRI. Such methods may eventually provide not only additional discrete resting networks but also better temporal information of tCS induced rsFC changes. Finally, we delivered tACS at theta frequency, but we did not take into account individual frequency bands and did not assess potential impact on frequency bands. Future work should include such assessment as previous work reported that theta oscillations correlated with the fronto-parietal network (Hacker et al., 2017). This is consistent with our observations that tACS applied at theta frequency increased rsFC between the left DLPFC and a key node of the fronto-parietal network (the right IPL) during and after the stimulation period. In sum, future tCS work combining neuroimaging methods such as fMRI and EEG will likely contribute at identifying stimulation parameters that will modulate relevant brain substrates to augment cognitive performance and alleviate symptoms.

## Data Availability Statement

The datasets generated for this study are available on request to the corresponding author.

## Ethics Statement

The studies involving human participants were reviewed and approved by University of Sherbrooke. The patients/participants provided their written informed consent to participate in this study.

## Author Contributions

SF and KW contributed to the conception and design of the study. SF, MM and CG collected data. MM, SG and ER performed the statistical analysis. MM wrote the first draft of the manuscript. All authors contributed to manuscript revision, read and approved the submitted version.

## Conflict of Interest

The authors declare that the research was conducted in the absence of any commercial or financial relationships that could be construed as a potential conflict of interest.

## Footnotes

^1^http://surfer.nmr.mgh.harvard.edu/
