# Supplementary material for: Effects of Transcranial Stimulation With Direct and Alternating Current on Resting-State Functional Connectivity: An Exploratory Study Simultaneously Combining Stimulation and Multiband Functional Magnetic Resonance Imaging
Source: Front Hum Neurosci. 2020 Feb 5;13:474. doi: 10.3389/fnhum.2019.00474 (PMC7012783; doi:10.3389/fnhum.2019.00474)
Supplement: Supplementary file 1 [file Data_Sheet_1.PDF]

Table 1. Significant interactions of resting-state functional connectivity and post-hoc comparisons for each significant cluster.

	Significant interactions from 2 (active, sham) × 4 (time points) repeated ANOVAs				Post-hoc paired t-tests between active and sham tCS from pre-tCS at each time point					
	**Cluster region (BA)**	**Cluster size**	**Peak MNI coordinates**	**Peak value**	**First 15 min of tCS**		**Last 15 min of tCS**		**After tCS**	
			**x, y, z**	**F**	**T**	***p***	**T**	***p***	**T**	***p***
**tDCS vs sham**										
L DLPFC seed	L IPL/SPL (BA 7/40)	168	-46, -60, 46	10.29	5.27	<. 001	4.97	<. 001	5.86	<. 001
	R IPL/SPL (BA 7/40)	129	38, -67, 53	7.45	5.43	<. 001	3.99	.002	4.57	.001
R DLPFC seed	*No significant cluster*									
**tACS vs sham**										
L DLPFC seed	R IPL (BA 40)	189	63, -39, 35	8.46	3.42	.008	2.72	.024	5.98	<. 001
R DLPFC seed	*No significant cluster*									

BA: Brodmann area; R: Right; L: Left; DLPFC: dorsolateral prefrontal cortex; IPL: inferior parietal lobule; SPL: superior parietal lobule.
